# The impact of CHIP premium increases on insurance outcomes among CHIP eligible children

**DOI:** 10.1186/1472-6963-14-101

**Published:** 2014-03-03

**Authors:** Silviya Nikolova, Sally Stearns

**Affiliations:** 1 Centre for Health Economics, Institute of Population Health, University of Manchester, Manchester, UK; 2 Department of Health Policy and Management, University of North Carolina at Chapel Hill, Chapel Hill, USA

**Keywords:** Health insurance, Premium, Health care reform

## Abstract

**Background:**

Within the United States, public insurance premiums are used both to discourage private health policy holders from dropping coverage and to reduce state budget costs. Prior research suggests that the odds of having private coverage and being uninsured increase with increases in public insurance premiums. The aim of this paper is to test effects of Children’s Health Insurance Program (CHIP) premium increases on public insurance, private insurance, and uninsurance rates.

**Methods:**

The fact that families just below and above a state-specific income cut-off are likely very similar in terms of observable and unobservable characteristics except the premium contribution provides a natural experiment for estimating the effect of premium increases. Using 2003 Medical Expenditure Panel Survey (MEPS) merged with CHIP premiums, we compare health insurance outcomes for CHIP eligible children as of January 2003 in states with a two-tier premium structure using a cross-sectional regression discontinuity methodology. We use difference-in-differences analysis to compare longitudinal insurance outcomes by December 2003.

**Results:**

Higher CHIP premiums are associated with higher likelihood of private insurance. Disenrollment from CHIP in response to premium increases over time does not increase the uninsurance rate.

**Conclusions:**

When faced with higher CHIP premiums, private health insurance may be a preferable alternative for CHIP eligible families with higher incomes. Therefore, competition in the insurance exchanges being formed under the Affordable Care Act could enhance choice.

## Background

The Children Health Insurance Program (CHIP) in the Unites States was created by the Balanced Budget Act of 1997 to help expand health insurance coverage to children in families with incomes which are too high to qualify for Medicaid, the public insurance program for individuals with low incomes. Although partly funded by the federal government, CHIP is administered by the states. States have flexibility in designing their cost-sharing and plan benefits structure, as well as in eligibility and enrollment provisions, subject to federal requirements. Most CHIP programs charge premiums to families based on income.

Public insurance premiums both discourage private health policy holders from dropping coverage and reduce state budget costs. Previous studies examined the effect of higher public insurance premium on public enrollment, private enrollment, and uninsurance rates [1,2]. The studies showed that the odds of having private coverage and being uninsured both increased with premium increases. Three other assessments showed that many children leaving state CHIP coverage lost any form of insurance [3-5]. Other studies documented a drop in health insurance coverage in response to rising premiums using different data sets [6-8].

Premium contributions for CHIP are influenced by the perceptions of legislators, the level of premium contribution required for public premium schedules in other states, the political context in which they were introduced, and the decisions by individuals regarding insurance purchase [9]. These considerations may have important bearing on the impacts of initial premium levels as well as premium increases over time [5]. The evaluation of premium effects on insurance purchase also must address selection in “enrollment” choice. Children with public or private health insurance may be different in terms of their family income, health, or preferences from children who are not insured [10].

We contribute to the existing literature by using a different research design (regression discontinuity (RD)) and a natural experiment due to the fact that children just below and above a state income cut-off are likely very similar in terms of observable and unobservable characteristics except the premium contribution, with children in families above the income cut-off having a higher premium. We used this jump in premium rate to evaluate the impact of CHIP premium on insurance outcomes using a regression discontinuity approach applied to the 2003 panel of the Medical Expenditure Panel Survey (MEPS) linked to premium and eligibility data at the state level.

Up until 2003, states rarely imposed cost sharing in their CHIP programs [11], but budgetary difficulties reversed this pattern when eleven states increased their premiums in 2003; in addition, one state imposed new cost sharing on the poorest families which did not have to pay such premiums before. We exploited the cross-sectional and temporal variation in premiums in 2003 to study the relationship between CHIP premiums and insurance outcomes.

## Methods

### Analytical methods

This paper measures the effect of variation in premium across individuals, states, and over time on insurance coverage. The cut-off rule for assigning CHIP eligible children to premium groups varies across states. We use “premium group” to denote a group of families with children who, based on state-specific income and age eligibility rules, fall into a CHIP premium bracket. We evaluate enrollment in the lowest two premium groups because of the small number of observations in higher premium groups. The income cut-off between premium groups is based on percentage of the federal poverty line (FPL) and varies across states to reflect local cost of living and budget availability. However, in every state with a two tier premium structure, the underlying principle is the same: for a given child age, families with family income *I* below a state specific income cut-off pay a smaller CHIP premium compared to families with income at or above the cut-off. Thus, the premium payment, as a function of *I*, contains a jump at the income cut-off between the premium groups. This discontinuity in premium payments fits within the conceptual framework of the Regression Discontinuity design. The method assumes threshold randomization, implying that, within small vicinity around the cut-off, children in the low and high premium groups near the cut-off are the same in terms of all observable and unobservable characteristics except the premium payment. The RD method has been shown to identify mean treatment effects for a subgroup of the population without having to rely on arbitrary assumptions about functional form and exclusion restrictions [12].

Since MEPS (described in the next section) is designed to produce nationally representative estimates, we first evaluate data on all states with a two tier premium structure as of January 2003. Premium level P_i_ varies across premium groups within a state and across states. The cross-sectional RD equation is defined as:

$$\;y_{\mathrm{is}}=\sum_{s\in S^{*}}\alpha_{s}D_{s}+\left(\beta_{0}+\beta_{p}\;P_{i}^{s}\right)1\left(I_{i}\geq {\bar{I}}^{s}\right)+g\left(I_{i},{\bar{I}}^{s}\right)+u_{\mathrm{is}},\;i\in {ℑ}^{s}$$

where *y*_*is*_ is the insurance enrollment outcome for child *i* in state s. S^*^ is the group of states with two premium groups. $1\left(I_{i}\geq {\bar{I}}^{s}\right)$ is an indicator function denoting assignment of child *i* to the high premium group in state *s*. ℑ^*s*^ denotes the subsample included in the estimation such that ${\bar{I}}^{s}-h<I_{i}<{\bar{I}}^{s}+h$ where *h* is the interval around the cut-off. The coefficient on the high premium group indicator is of primary interest and is defined as: $\;\beta_{0}+\beta_{p}P_{i}^{s}$. This linear function captures the change in insurance status in response to premium change. The parameter *β*_0_ captures the average enrollment for children above the income threshold level, and *β*_*p*_ is the linear effect of the premium on the likelihood a child is insured. We control for state-specific fixed effects with a set of dummies *D*_*s*_. The state dummies allow low premium groups to be different across states, while the high premium groups can differ only in $\left(\beta_{0}+\beta_{p}\;P_{i}^{s}\right)$. Since income *I* is the only systematic determinant of the premium fee and income influences insurance choice [13,14], the inclusion of a smooth function $g\left(I_{i},{\bar{I}}^{s}\right)$ which is continuous at the state income cut-off ${\bar{I}}^{s}$ solves the endogeneity issue [12]. Thus, after controlling for differences in premium and income and for state-specific fixed effects, we presume no other factors affect the insurance outcomes of children. We therefore estimate a “sharp” RD model since the “simulated” insurance status is a deterministic function of family income.

We apply a Difference-in-Differences (DD) method to states with a two tier premium structure as of December 2003 to evaluate the impact of premium increases over time:

$$\begin{array}{l} y_{\mathrm{ist}}=\sum_{s\in S^{**}}\alpha_{s}D_{s}+\left(\beta_{0}+\beta_{1}\;P_{\mathrm{it}}^{s}\right)1\left(I_{\mathrm{it}}\geq {\bar{I}}_{t}^{s}\right)\\ \;+\left(\gamma_{0}+\gamma_{1}\;P_{\mathrm{it}}^{s}\right)t+\left(\delta_{0}+\delta_{1}\;P_{\mathrm{it}}^{s}\right)\left(t*1\left(I_{\mathrm{it}}\geq {\bar{I}}_{t}^{s}\right)\right)\\ \;+\beta_{z}^{'}z_{\mathrm{ist}}+u_{\mathrm{ist}},\;i\in {ℑ}^{s} \end{array}$$

where S^**^ is the relevant group of states. *y*_*ist*_ is the insurance enrollment status of child *i* in state *s* at time *t*. ℑ^*s*^ is the subsample included in the estimation. The state dummies (*D*_*s*_) control for state heterogeneity. Premium, time and premium-time interactions are modelled as linear specifications. The set of premium variables *β*_1_, *γ*_1_  and *δ*_1_ capture the linear premium effect for being in the high premium group, for being in the second period, and their interaction. The variables *β*_0_, *γ*_0_ and *δ*_0_ capture, respectively, the average enrollment for the high premium groups, in the post premium increase period, and for high premium groups in the second period. ^*z*^ is a set of other variables that affect insurance status.

We provide summary statistics on child age and health for the groups above and below the income cut-off and compare their means using data on the largest state in our data set to evaluate whether RD design is appropriate for the purposes of our study.

### Data

The data for the analysis come from the 2003 MEPS panel. MEPS is designed to produce nationally representative estimates for insurance coverage, medical expenditure, and health care use. It provides detailed data on a wide range of health, demographic, and socioeconomic characteristics [15]. We collected data on CHIP premiums and eligibility from program websites for all states and the District of Columbia. The premium data were merged by state to the 2003 full-year consolidated MEPS files. Ethical approval has been obtained from the Institutional Review Board (study number 05–0944 at the University of North Carolina at Chapel Hill).

As of January 2003, 18 CHIP programs with at least a two tier premium structure charged families up to $61 per child per month if in the low premium group and up to $77 if in the high premium group. Eleven states increased premiums over the course of 2003. By December 2003, an additional state adopted a two-tier premium structure, so these 19 states are used in the longitudinal DD analysis.

Each state’s premium information is used to assign the premium amount that the family unit will face to cover one child for one month. We have not included in our analysis states that charge annual premiums as we seek to evaluate the impact of monthly CHIP premium contributions on insurance status. The longitudinal sample is further constricted to include only children who had positive full year weights for 2003 and participated in MEPS for the entire year. We evaluate January enrollment outcomes for CHIP eligible children in the cross-sectional analysis. For our longitudinal analysis, we focus on January and December. In addition to premium, we control for family income, health and age of the child which are obtained from MEPS. Health is verified by asking parents whether the child gets sick easily, with higher scores pointing to better child health. Child age is measured as of the end of 2003.

### Assigning program eligibility and insurance status

The analysis relies on CHIP eligibility simulation that predicts the eligibility status of each child in each state using information on family income, family structure, child age, and state-specific eligibility rules as they would be applied to new applicants. If a child is assigned CHIP eligibility and is observed in the data to have public insurance, CHIP insurance status is assigned. A CHIP eligible child observed in the data to hold private health insurance is assigned private insurance status. Uninsurance status is assigned if a CHIP-eligible child is not recorded to have public or private insurance coverage. Since CHIP eligibility is determined on monthly basis, the data incorporate the possibility of a change in insurance status due to changes in state eligibility rules and/or premium payments and/or child age. An online Additional file 1 provides details of the assignment.

## Results

### Summary statistics

Table 1 presents descriptive statistics for children in low and high premium groups in 18 states with a two tier premium structure as of January 2003. Children in the first income group are, on average, slightly older than children in the second group. Family income, by construction, increases with premium group. CHIP enrollment is lower, private insurance take-up is higher, and the uninsurance rate is lower among children in the high versus low premium group.

**Table 1:** Summary statistics -- cross-sectional data

		**Mean**	**St.d.**
Low premium group (n = 587)	Insurance		
	CHIP	0.52	0.5
	Private	0.269	0.444
	Uninsured	0.213	0.41
	Income (% FPL)	126.2	14.88
	Premium	5.02	5.52
	Child age	10.97	4.36
High premium group (n = 1,044)	Insurance		
	CHIP	0.303	0.4
	Private	0.576	0.494
	Uninsured	0.122	0.327
	Income (%FPL)	189.9	26.52
	Premium	12.66	10.31
	Child age	9.82	5.1

Characteristics for children in low premium and high premium groups in 18 states with a two tier premium structure, January 2003.

Table 2 shows descriptive statistics for children in low premium and high premium groups in 19 states with a two-tier premium structure as of January 2003 and December 2003. For both premium groups, average family income, child age and health remain relatively constant over the course of the year, and average premium increases. Unadjusted rates for public and private insurance and uninsurance remain similar over 2003.

**Table 2:** Summary statistics – longitudinal data

		**January**			**December**		
		**Mean**	**St.d.**	**N**	**Mean**	**St.d.**	**N**
Low premium group	CHIP	0.423	0.494	926	0.42	0.494	932
	Private	0.367	0.482		0.368	0.483	
	No insurance	0.217	0.412		0.216	0.411	
	Income (% FPL)	142.75	43.27		143.03	43.35	
	Premium ($)	4.99	7.17		5.88	7.16	
	Child age	10.16	4.63		10.15	4.65	
	Health state*	3.49	1.64		3.48	1.65	
High premium group	CHIP	0.33	0.47	604	0.32	0.47	598
	Private	0.575	0.495		0.577	0.494	
	No insurance	0.101	0.302		0.1	0.301	
	Income (% FPL)	191.94	27.91		192.29	27.83	
	Premium($)	10.55	7.13		11.53	7.53	
	Child age	9.88	4.99		9.84	4.98	
	Health state*	3.47	1.69		3.46	1.7	

(*) - Health status is taken by the question whether the child gets sick easily where 1 stands for definitely true and 5 is for definitely not.

Characteristics for children in low premium and high premium groups in 19 states with a two tier premium structure, January and December, 2003.

### Cross-sectional analysis

Table 3 shows the cross-sectional RD estimates for the association between CHIP premium and insurance coverage. The results indicate that a $1 increase in the premium above the income cut-off is associated with a higher probability of being privately insured in the range of 2.2 to 1.7 percentage points (p < 0.05) for bandwidths of ±15% to ±25% of the Federal Poverty Level, respectively. Estimates for associations with public insurance and uninsurance status are not statistically significant. These findings suggest that child coverage through private insurance may become a preferable alternative for CHIP families in the higher income group when faced with higher CHIP premiums [16]. A test of the joint significance of the state-specific dummies shows statistically significant heterogeneity among the low premium groups across states in terms of insurance enrollment status. Additionally, a test of income significance shows that, when observations are close to the state-specific cut-off, family income does not have a statistically significant impact on the probability of public insurance, private insurance or uninsurance. This indicates that changes in these probabilities are captured by the other covariates in the regressions.

**Table 3:** Cross-sectional regression discontinuity estimates of a $1 increase in CHIP premium for three bandwidths presented as % of FPL using data on 18 states

**%**	**CHIP**	**Private**	**Uninsurance**	**Number of children above and below cut-off**
−15/+15	−0.014	0.022*	−0.008	212/167
	(.011)	(.010)	(.009)	
−20/+20	−0.009	0.018*	−0.009	256/191
	(.009)	(.009)	(.008)	
−25/+25	−0.007	0.017*	−0.010	317/230
	(.008)	(.008)	(.006)	

(*) - indicates significance at the 5 per cent confidence level.

### Longitudinal analysis

The first set of columns in Table 4 shows the difference-in-differences estimates for the impact of CHIP premium increases during 2003 on insurance coverage among CHIP eligible children. Our findings suggest that a $1 variation in increases in CHIP premiums over time are associated with a statistically significant increases in disenrollment from CHIP (range of 1.4 to 2.1 percentage points) and in private insurance take-up (range of 1.1 to 2.2 percentage points), but that CHIP premium increases have no effect on uninsurance across the bandwidths considered. The second set of columns reports the high premium group dummy estimates from the difference-in-differences estimation for different insurance types. These estimates show that children in the high premium group are statistically significantly more likely to be privately insured as well as statistically significantly less likely to be uninsured. Time invariant characteristics of the high premium group such as access to private insurance options and/or cost of these alternatives in terms of monetary (other than premium) and non-monetary dimensions can explain the higher probability of private insurance and lower probability of uninsurance. The remaining columns in Table 4 report the time dummy estimates. The time dummy captures the change in insurance coverage in response to time-varying factors that are not explicitly controlled for but are constant across states. In all three regressions, we find small and mostly statistically insignificant results for the time dummy, suggesting that other time-varying covariates have not contributed to the change in insurance status of CHIP eligible children in 2003. A joint test of income significance reveals that income is not a statistically significant determinant of the probability of CHIP coverage, private coverage, or uninsurance when children with family income near the state income cut-off are considered. A joint test of the statistical importance of state dummies shows that state specific factors are associated with the likelihood of public insurance and private insurance, but not uninsurance.

**Table 4:** Difference-in-differences estimates for three different bandwidths presented as % of FPL using data on 19 states

**%**	**Premium jump of $1**			**High premium group dummy**			**Time dummy**			**Number of children above and below the cut-off**
	**CHIP**	**Private**	**Uninsurance**	**CHIP**	**Private**	**Uninsurance**	**CHIP**	**Private**	**Uninsurance**	
−15/+15	−0.021*	0.022*	−0.004	0.028	0.240*	−0.331*	−0.063	−0.009	0.080	166/164
	(.005)	(.005)	(.005)	(.089)	(.080)	(.082)	(.046)	(.042)	(.042)	
−20/+20	−0.019*	0.016*	0.000	0.032	0.107	−0.202*	−0.030	−0.012	0.055	192/210
	(.004)	(.004)	(.004)	(.076)	(.070)	(.066)	(.042)	(.039)	(.037)	
−25/+25	−0.014*	0.011*	−0.001	0.005	0.189*	−0.232*	−0.064	−0.017	0.086*	230/250
	(.004)	(.003)	(.003)	(.072)	(.062)	(.061)	(.040)	(.035)	(.034)	

(*) - indicates significance at the 5 per cent confidence level.

### Testing of assumptions

We test the RD assumption that children just below and just above a state-specific income cut-off are similar in all observable and unobservable characteristics except the premium contribution. Table 5 shows the mean age and health of children in the largest state in our data set. The differences between the means are small. Results from a t-test show no statistically significant difference between the means from the two samples. This null finding suggests that the observed change in insurance enrollment at the cut-off is likely to be related to the jump in premium, and not to child age and health, even though previous literature has shown that insurance coverage is associated with child age and health [10].

**Table 5:** Summary statistics for age and health for the largest state

**Bandwidth**	**Group**	**Number of chidlren**	**Age**	**Health**
15	Low premium	88	9.227	3.648
			(5.13)	(1.576)
	High premium	109	9.364	3.618
			(5.037)	(1.49)
20	Low premium	98	9.143	3.755
			(5.007)	(1.554)
	High premium	130	9.331	3.654
			(4.937)	(1.418)
25	Low premium	130	9.1	3.746
			(5.031)	(1.464)
	High premium	150	9.367	3.627
			(4.966)	(1.44)

## Discussion

Prior evidence suggests that CHIP premium increases increase both the likelihood of being privately insured or uninsured; given this prior evidence, researchers have argued that premium schedules should be designed to preserve the accessibility of public insurance and prevent private insurance crowd-out [1,2,5,10]. This analysis adds to the existing literature by using a different research design (regression discontinuity) and a natural experiment due to the fact that children just below and above the cut-off are likely very similar in terms of observable and unobservable characteristics except the premium contribution. Specifically, we used data from 19 states and took advantage of a unique feature of the CHIP program which creates a discontinuity in premium, with families with income above a state-specific cut-off paying more for CHIP public insurance for their child. The cross-sectional analysis showed that a $1 increase in premium above the cut-off is associated with an increase in the probability of private insurance in the range of 1.7 to 2.2 percentage points for children with higher family income but had no impact on public insurance or uninsurance. The difference-in-differences results point to an increase in the probability of private insurance in between 1.1 and 2.2 percentage points and to a decline in the probability of CHIP enrollment in the range of 1.4 to 2.1 percentage points in response to premium increases of $1 over time. Neither analysis showed increases in the rate of uninsurance. These findings suggest that for CHIP eligible families with higher incomes, child coverage through private insurance may become a preferable alternative when faced with higher CHIP premiums. The results also underscore the importance to these families of keeping their children insured.

A potential limitation of our study is that we do not take into account the total premium spending at the family level or the caps on these payments imposed in some states. Premium costs for all children, as opposed to one child, represent a more sizeable share of family income, and insurance coverage decisions are likely made at the family level. Another limitation is that CHIP eligibility and insurance coverage are not directly observed and, therefore, must be imputed. Measurement error can result because the assignment procedure fails to completely replicate the “true” process or simply because income information has been misreported.

## Conclusion

The relationships identified in this analysis, especially the longitudinal estimates, are potentially informative regarding the effects of premium increases for CHIP. The results may also be relevant for state health insurance exchanges as the provisions of the Affordable Care Act are implemented. Increases in public insurance premiums can cause families to switch to private insurance options, such as plans that will be available through state health insurance exchanges. The ability of exchanges to offer insurance at competitive prices can enhance both choice and efficiency in insurance plan selection. Yet even in the face of CHIP premium increases, families do not seem to switch to a state of uninsurance, at least in the group of families eligible for CHIP.

## Competing interests

The authors declare that they have no competing interests.

## Authors’ contributions

SN collected and prepared data for analysis, performed statistical analysis, and contributed to the design of the study. SS contributed to the design of the study. Both authors analysed results, participated in the writing, and reviewed the final manuscript. Both authors read and approved the final manuscript.

## Pre-publication history

The pre-publication history for this paper can be accessed here:

http://www.biomedcentral.com/1472-6963/14/101/prepub

## Supplementary Material

Additional file 1 Eligibility and Insurance Status Assignment. Click here for file
