# Supplementary material for: The impact of CHIP premium increases on insurance outcomes among CHIP eligible children
Source: BMC Health Serv Res. 2014 Mar 3;14:101. doi: 10.1186/1472-6963-14-101 (PMC3946024; doi:10.1186/1472-6963-14-101)
Supplement: Additional file 1 — Eligibility and Insurance Status Assignment. [file 1472-6963-14-101-S1.pdf]

Appendix: Eligibility and Insurance Status Assignment

We have monthly data on child age and insurance status as well as monthly data on state premium levels and eligibility rules. Family income is ascertained on an annual basis in the Medical Expenditure Panel Survey (MEPS) data.

We use the Health Insurance Eligibility Unit (HIEU) definition when determining eligibility. This variable identifies family members who would normally be eligible for family coverage under the adults' private health insurance family plans. These families, or HIEUs, comprise adults, their spouses, and their unmarried natural/adoptive children aged 18 and under. For traditional families with parents and children, we calculate annual family income by summing up the annual wage and salary income for each adult in the HIEU. If young adults aged 18-24 in an HIEU are full-time students, their income is omitted from the family income calculation. However, if the student is a parent and not an older sibling, their income is counted towards the calculation of family income. All members in the HIEU, including Medicaid-eligible children, are counted in the determination of family income as a percentage of the federal poverty line. In many states continuous eligibility is granted for 6 months or more and income in the past month may not track with the family's income level at the time that eligibility was determined.

Unmarried minors not living with their natural/adoptive parents are included in the family of their stepparent, grandparent, or aunt/uncle. State rules vary for the counting of income and family size for these "non-traditional" families. To simplify, following previous MEPS studies that use simulated eligibility [1, 2], we assume that all non-traditional guardians who are both low-income and disabled are included in the unit for both income and size, which

increases the likelihood of being eligible. Otherwise, the family is treated as a child-only case and the child is eligible for public insurance despite the income of the adults.

We use the Urban Institute Welfare Rules Database to determine which states allow minor parents to head their own household. For these states, we follow rules similar to those for non-traditional families: parent/adult relatives are included only if they were both low-income and disabled. Otherwise the minor parent heads their own household. For states that do not allow minor parents to head their own household, parent/adult relatives are included in the family unit for income and size.

References

[1] J. Hudson, T. Selden and J. Banthin, “The Impact of SCHIP on Insurance Coverage of Children,” *Inquiry*, vol. 42, pp. 232-254, 2005.

[2] J. Hudson, J. Banthin and T. Selden, “Tracking changes in Eligibility and Coverage Among Children 1996-2002,” *Health Affairs*, vol. 23, no. 5, pp. 39-50, 2004.
